# Baseline new bone formation does not predict bone loss in ankylosing spondylitis as assessed by quantitative computed tomography (QCT) - 10-year follow-up

**DOI:** 10.1186/1471-2474-12-121

**Published:** 2011-05-31

**Authors:** Mariusz Korkosz, Jerzy Gąsowski, Piotr Grzanka, Janusz Gorczowski, Wojciech Pluskiewicz, Sławomir Jeka, Tomasz Grodzicki

**Affiliations:** 1 Division of Rheumatology, Department of Internal Medicine and Gerontology, Jagiellonian University, Sniadeckich 10, Krakow, 31-531, Poland; 2 Department of Internal Medicine and Gerontology, Jagiellonian University, Sniadeckich 10, Krakow, 31-531, Poland; 3 Imaging Unit, Department of Medicine, Jagiellonian University, Skawinska 8, Krakow, 31-066, Poland; 4 Densitometry Unit, Malopolskie Centrum Medyczne, Rejtana 2, Krakow, 30-510, Poland; 5 Metabolic Bone Diseases Unit, Medical University of Silesia, 3-Maja 13-15, Zabrze, 41-800, Poland; 6 Department of Rheumatology and Connective Tissue Diseases, 2nd University Hospital, Ujejskiego 75, Bydgoszcz, 85-168, Poland; 7 Department of Internal Medicine and Gerontology, Jagiellonian University, Sniadeckich 10, Krakow, 31-531, Poland

## Abstract

**Background:**

To evaluate the relationship between bone loss and new bone formation in ankylosing spondylitis (AS) using 10-year X-ray, dual-energy x-ray absorptiometry (DXA) and quantitative computed tomography (QCT) follow-up.

**Methods:**

Fifteen AS patients free from medical conditions and drugs affecting bone metabolism underwent X-ray, DXA and QCT in 1999 and 2009.

**Results:**

In spine QCT a statistically significant (p = 0,001) decrease of trabecular bone mineral content (BMC) was observed (change ± SD: 18.0 ± 7.3 mg/cm^3^). In contrast, spine DXA revealed a significant increase of bone mineral density (change ± SD: -0.15 ± 0.14 g/cm^2^). The mean BMC, both at baseline and follow-up was significantly lower (p = 0.02 and p = 0.005, respectively) in advanced radiological group as compared to early radiological group. However, in multiple regression model after adjustment for baseline BMC, the baseline radiological scoring did not influence the progression of bone loss as assessed with QCT (p = 0.22, p for BMC*X-ray syndesmophyte scoring interaction = 0.65, p for ANOVA-based X-ray syndesmophyte scoring*time interaction = 0.39). Baseline BMC was the only significant determinant of 10-year BMC change, to date the longest QCT follow-up data in AS.

**Conclusions:**

In AS patients who were not using antiosteoporotic therapy spine trabecular bone density evaluated by QCT decreased over 10-year follow-up and was not related to baseline radiological severity of spine involvement.

## Background

**Figure 1:**
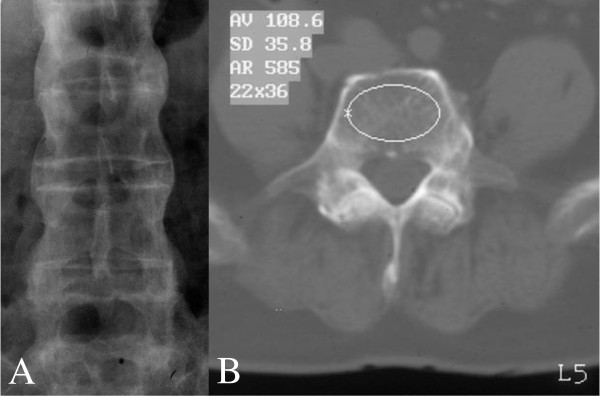
**(A) Anteroposterior lumbar radiograph with advanced syndesmophytes and (B) Quantitative Computed Tomography scan of L5 vertebral body showing elliptical region of interest (ROI) containing trabecular bone (48-year-old patient at baseline)**.

Osteoporosis is a well recognized early feature of ankylosing spondylitis (AS) particularly pronounced in active disease [1,2]. In AS axial osteoporosis coexists with new bone formation, ie. syndesmophytes, spine ligaments ossification and facet joints ankylosis, thus the outer layer of the spine is hipermineralized (Figure 1A) as opposed to bone density within vertebral bodies which is diminished. In the effect the risk of vertebral fractures increases [3,4].

Bone density in AS is assessed by dual-energy x-ray absorptiometry (DXA) and quantitative computed tomography (QCT). The DXA measures areal bone mineral density (BMD, g/cm^2^). Spine anteroposterior DXA reliably detects osteoporosis in early cases but in advanced AS it overestimates BMD due to new bone formation. Spine QCT facilitates measurement of volumetric trabecular bone mineral content (BMC, mg/cm^3^) with no cortical bone and "outer layer" artifacts. The discrepancy between DXA and QCT in AS is marked in advanced disease, where the majority of patients have normal DXA BMD but QCT shows trabecular bone loss [5].

AS patients with more pronounced new bone formation are at greater risk of osteoporosis [6]. However, to-date there is paucity of prognostic data to predict this serious complication.

Therefore the objective of this study was to evaluate the relationship between bone loss and new bone formation in AS using 10-year X-ray, DXA and QCT follow-up.

## Methods

### 1. Patients

In 1999 thirty seven males fulfilling New York modified AS criteria, with no concomitant diseases and drugs affecting bone metabolism, underwent X-ray (spine), DXA (spine and hip) and QCT (spine) measurements [7]. In 2009 fifteen patients underwent X-ray, DXA and QCT. Of the 22 excluded patients, 14 were treated with bisphosphonates and/or corticosteroids, 3 refused to participate, 1 lost contact. Three patients died. In one patient QCT was not feasible due to unsatisfied positioning.

### 2. Imaging and bone density

We obtained standard radiographs of the lumbar and thoracic spine at baseline and follow-up.

DXA was performed at L2-L4 and left hip using Lunar DPX-IQ (1999) and Lunar Prodigy (2009, Lunar, USA). Right hip was scanned if the left was difficult to position or hip replacement had been performed. In one patient with new L2 and L4 fractures L3 was analyzed. The coefficient of variation (CV) for repeated measurements in vivo and least significant change at 95% confidence level (spine) were 2,2% and 0,062 g/cm^2 ^for DPX-IQ and 1,6% and 0,043 g/cm^2 ^for Prodigy, respectively. T-scores and g/cm^2 ^were based on manufacturer-supplied reference values.

QCT (L1-L5) was determined at baseline and follow-up with single-energy QCT (Twin Flash, Marconi, USA). Standard calibration phantom (Picker) recommended by Marconi was used for each scan. First, from the lateral lumbar spine scannogram the midportions of L1-L5 vertebral bodies parallel to the endplates were localized manually. Then, scans of 10 mm thickness were carried out in each vertebrae and other scanning parameters were set according to scanner manufacturer. Data from scanning elliptical region of interest (ROI, Figure 1B) including only trabecular bone, was analyzed using software CirsCT Bone Densitometry of CIRS company supplied by scanner manufacturer. T-scores were calculated using manufacturer's reference data. The fractured vertebrae were excluded from analyses.

### 3. Statistical analysis

We compared means using t-test and proportions using chi-square test, where applicable. To check the prognostic value of studied variables on follow-up BMC, we fitted multiple regression models, both without and after adjustment for baseline values of age, body mass index (BMI), erythrocyte sedimentation rate (ESR), smoking, QCT derived BMC stratum, radiologic score and duration of disease prior to baseline visit. To enable visual inspection of individual patient data, we plotted the baseline and follow-up values of measures of bone-density in strata of baseline BMC based on QCT. To formally test the between-subject (BMC baseline strata) differences in studied variables, we fitted the two-way ANOVA models. Finally, using linear regression approach, we assessed interaction between BMC and radiographic scoring at baseline, in their influence on measures of bone density at follow-up.

## Results

### 1. Patient characteristic

Mean ± SD baseline age of 15 included patients was 45.6 (± 7.3) years and disease duration averaged 16.5 (± 8.6) years. ESR averaged 41.8 mm/hr (± 27.5), BMI 27.4 (± 4.2), 33,3% patients were smoking. Three patients experienced four new vertebral fractures during follow-up (Th7, Th12, L2, L4). Baseline and follow-up densitometry results are shown in table 1.

**Table 1:** Mean DXA and QCT results at baseline and at follow-up.

	baseline		follow-up		change	p
	**absolute**	**T-score**	**absolute**	**T-score**		

QCT L1-L5, mean (mg/cm^3^)	94.2	-3.654	76.1	-4.468	18.0	0.001*

(SD)	31.8	1.246	33.5	1.499		

median (25th-75^th ^percentile)	96.5 (68.2-122.5)		77.5 (47.0-113.7)			

DXA L2--L4, mean (g/cm^2^)	1.027	-1.780	1.180	-0.493	-0.15	0.0009*

(SD)	0.183	1.531	0.198	1.649		

median (25th-75^th ^percentile)	0.96 (0.86-1.24)		1.19 (1.0-1.35)			

DXA neck, mean (g/cm^2^)	0.892	-1.131	0.926	-1.108	-0.03	0.48

(SD)	0.134	1.077	0.279	2.139		

median (25th-75^th ^percentile)	0.86 (0.83-0.92)		0.88 (0.83-0.96)			

DXA Wards, mean (g/cm^2^)	0.737	-1.715	0.727	-1.792	0.01	0.81

(SD)	0.129	0.992	0.231	1.776		

median (25th-75^th ^percentile)	0.73 (0.65-0.82)		0.69 (0.62-0.79)			

DXA, dual-energy x-ray absorptiometry; QCT, quantitative computed tomography.

### 2. Relationship between QCT and DXA results

**Figure 2:**
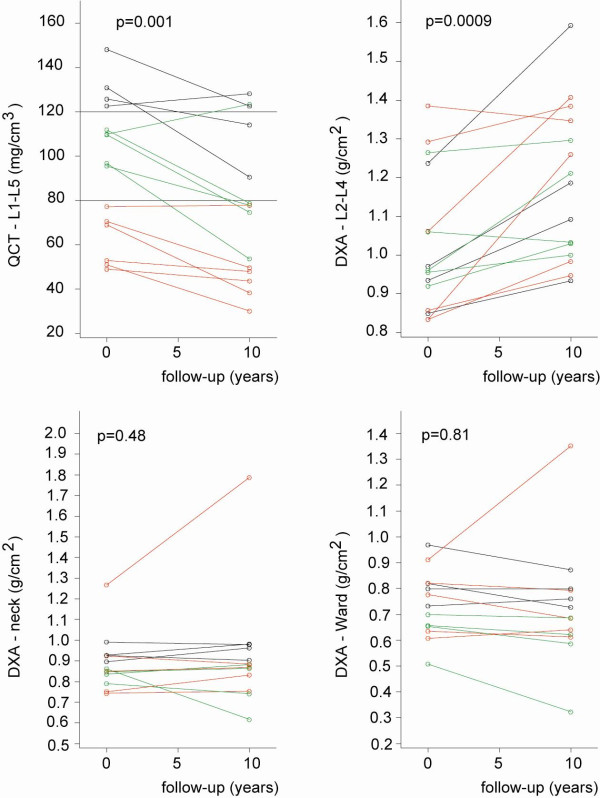
**Measures of bone density at baseline and after 10-year follow-up in 15 patients in four regions of interest: L1-L5 (QCT), L2-L4 (DXA), neck (DXA) and Wards (DXA) with regard to BMC baseline stratification**. Each baseline BMC stratum is represented by different colors: black (normal), green (osteopenia), red (osteoporosis).

We used stratification of patients based on baseline QCT according to Kalender et al. [8](normal >120; osteopenia 80--120; osteoporosis < 80; mg/cm^3^) to follow changes in QCT and DXA. In QCT at baseline there were 4 patients in normal stratum, 5 had osteopenia and 6 osteoporosis. To follow changing bone density data were plotted for individual patients (Figure 2). In spine QCT a statistically significant (p = 0,001) decrease of bone density (change ± SD: 18.0 ± 7.3) was observed, and it was universal across all strata (Figure 2). There were no clear-cut trends across strata in DXA that would resemble those noted in spine QCT. In spine DXA, a significant BMD change was noted (p = 0,0009) with trend towards increased density (change ± SD: -0.15 ± 0.14). Neither neck nor Ward DXA changed from baseline to follow-up. There was no correlation between QCT and DXA, both in density units and T- scores - at baseline and follow-up. Additionally, to test the between-subject (BMC baseline strata) differences in studied variables, we fitted the two-way ANOVA models. We found that the baseline stratification by BMC significantly influenced the BMC values both at baseline (p < 0.0001) and at follow-up (p = 0.0009), but had no impact on the indices derived from DXA (all p > 0.07). We found no interaction between baseline BMC stratum and time-related change in all studied variables (all p > 0.31).

### 3. Relationship between QCT and new bone formation

**Figure 3:**
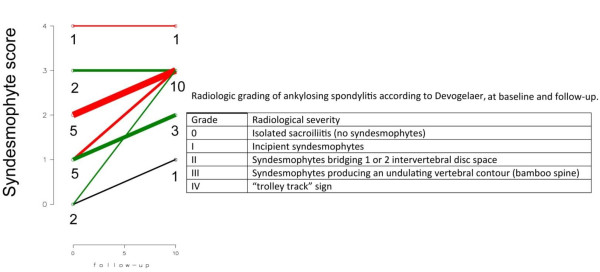
**Radiographic grading at baseline and follow-up according to Devogelaer **[9]. Digits represent number of patients in each grade at baseline and follow-up. Thickness of lines is proportional to numbers of patients. Embodied is a Devogelaer X-ray grading.

To determine the effect of radiological severity on BMD/BMC change X-rays were assessed and patients were split into two groups based on baseline syndesmophyte scores according to Devogelaer [9] (table embedded in Figure 3); early group (grade 0-I, ie. no definite syndesmophytes) - 7 patients; and advanced group (grade II-IV, ie. bridging syndesmophytes) - 8 patients (Figure 3). At follow-up 6 patients progressed into advanced group.

The mean BMC, both at baseline and follow-up was significantly lower (p = 0.02 and p = 0.005, respectively) in advanced group (77.2 ± 21.5 and 55.4 ± 18.9 mg/cm^3^, baseline and follow-up, respectively) as compared to early group (113.6 ± 31.7 and 99.8 ± 31.1 mg/cm^3^, baseline and follow-up, respectively).

However, in multiple regression model after adjustment for baseline BMC, the baseline radiological scoring did not influence the progression of bone loss as assessed with QCT (p = 0.22, p for BMC*X-ray syndesmophyte scoring interaction = 0.65, p for ANOVA-based X-ray syndesmophyte scoring*time interaction = 0.39).

To discover the predictive value of other variables possibly influencing 10-year changes in BMC similar multiple regression model was used. Only baseline QCT values (mg/cm^3^) predicted 10-year decrease in QCT; with no influence of baseline DXA values, disease duration, age, ESR, smoking and BMI.

## Discussion

We showed that in patients with ankylosing spondylitis who were free from medical conditions and drugs affecting bone metabolism, spine trabecular bone density evaluated by QCT decreased over 10-year follow-up. Despite of lower BMC in patients with more advanced radiology scoring, the 10-year change was not related to baseline radiological severity of spine involvement.

Study population was highly homogeneous and included men with no history of steroid or bisphosphonate use over 10-year follow-up. The baseline syndesmophyte formation was equally distributed between early and advanced disease. Thus we have had a group of early new bone formation, ie. scores 0--I corresponding to lack or mild syndesmophyte formation and advanced new bone formation, ie. scores II--IV corresponding to severe bridging osteoproliferation. The higher radiology scores in Devogelaer grading (Figure 3) the more pronounced redistribution of bone, ie. loss of vertebral bodies trabecular bone density is accompanied by an increase in density of the outer layer of spine. This phenomenon seems to be responsible for higher vertebral fracture risk in AS since the osteoporosis within vertebral bodies associated with increased rigidity of spine makes it more vulnerable to fractures. The evolution of these two contrary processes in the natural history of AS is responsible for axial not peripheral fractures [10,11]. There is an evidence that different bone density measurements, including spine QCT showed similar percentage differences (range 16--22%) between subjects with vertebral fractures and controls [12-14] but in AS these differences have not been examined yet.

We showed that QCT is a reliable technique and spine a correct region to follow-up long term changes in BMC, reflecting natural history of osteoporosis in ankylosing spondylitis (Figure 2). Decline in BMC was statistically significant and neither DXA of spine nor femoral neck or Wards revealed a similar trend. In our group, one patient showed an outlying increase in neck and Ward's values (Figure 2). After exclusion of this individual the p value for change in DXA neck measurement did not change (p = 0.80). However we observed a small yet statistically significant (p = 0.02) decrease in DXA measurement of Ward's triangle which represents trabecular bone and is compatible with our QCT findings. This observation strengthens the possible impact of our message, that the measurement of trabecular bone (either QCT or DXA Ward's triangle) should replace integral measurement of cortical and trabecular bone (DXA neck or spine) in assessment of AS patients.

In addition, regression analysis determined that the only one significant predictor of the final BMC was baseline BMC. Lee did not show significant changes in BMC over 15-month period, to date the longest published study with QCT in AS [15]. They followed 14 patients; 7 with sacroiliitis alone (early AS) and 7 with extensive vertebral syndesmophytes (late AS) thus with number and strata resembling our group.

Our results suggest that baseline syndesmophytes score has impact on trabecular bone density as early and advanced groups differ regarding QCT values at baseline and follow-up. Karberg using QCT and DXA reported that greater bone loss is detected more frequently in patients with syndesmophytes [6] and our results confirm this as mean BMC in our advanced group was in osteoporosis range both at baseline and at follow-up. In contrast mean BMC in early group was within osteopenia range, both at baseline and follow-up.

Although early and advanced groups differ in QCT values, the baseline radiological severity had no predictive value for bone loss over 10 years. It thus seems likely that in ankylosing spondylitis bone loss does not parallel new bone formation, contrary to findings by Karberg et al. [6].

Our study has to be considered within context of its limitations. The sample-size is relatively low, which restricted our use of statistical techniques to disentangle the effect of possible confounders. The NSAIDs treatment, both continuous and on demand, may have influenced syndesmophyte growth. It is hard to separate the influence of disease and aging on trabecular bone density as well. However, ours is longest follow-up data available in a highly homogenous group of patients. Despite of the relatively small group, we had > 80% power to detect 20% difference in the BMC from baseline to follow-up, with the 5% significance. On the other hand, we are aware, that due to moderately-sized sample size the results we obtained must be considered with caution.

Intriguing question is whether there is a link between bone resorption and new bone formation in AS or whether inflammation triggers both processes. Bone loss is already seen in early AS and is due to disturbed bone turnover rather than immobility caused by syndesmophytes [6,9,15-17]. Markers of bone resorption were found to be positively correlated with ESR and CRP [18-20]. TNF blockade decreased inflammation in AS and increased BMD [21], observations which link inflammation with bone resorption.

Interaction between new bone formation and inflammation in AS is not clear. Osteocalcin was found to be decreased [22], normal [15,20] or increased [23] in AS and was unrelated to levels of inflammatory markers. Most syndesmophytes are not associated with inflammation as based on MRI studies [24]. Anti-TNF treatment in spondyloarthritis increased bone-specific alkaline phosphatase (BALP) and negative correlation between BALP and metalloproteinase-3 (bone destruction marker) was discovered, indicating that new bone formation in AS occurs if inflammation is depressed [25]. Thus it seems likely that bone loss and new bone formation in AS are not directly linked with inflammation, and according to our data they are not coupled - a hypothesis which requires further studies.

## Conclusions

In AS patients who were not using antiosteoporotic therapy spine trabecular bone density evaluated by QCT decreased over 10-year follow-up and was not related to baseline radiological severity of spine involvement.

## Abbreviations

AS: ankylosing spondylitis; DXA: dual-energy x-ray absorptiometry; QCT: quantitative computed tomography; BMD: bone mineral density; BMC: bone mineral content; ROI: region of interest; CV: coefficient of variation; ESR: erythrocyte sedimentation rate; CRP: C-reactive protein; BMI: body mass index; BALP: bone-specific alkaline phosphatase; TNF: tumor necrosis factor; NSAIDs: nonsteroidal anti-inflammatory drugs.

## Competing interests

The authors declare that they have no competing interests.

## Authors' contributions

MK was responsible for study concept and design, patients assessment, analysis of investigated data and its interpretation, and drafting manuscript. JG performed statistical analysis, participated in data analysis and interpretation, figures preparation and drafting manuscript. PG was responsible for QCT scanning and analysis, and quality control of data. JG carried out DXA scanning and analysis, and quality of data. WP participated in study concept and design, DXA and QCT quality control, and data interpretation. SJ was involved in analysis of X-ray, DXA and QCT data, and statistical analysis. TG was responsible for study concept and revising manuscript. All authors read and approved the final manuscript.

## Pre-publication history

The pre-publication history for this paper can be accessed here:

http://www.biomedcentral.com/1471-2474/12/121/prepub
